# Nanoemulsion and Nanogel Containing *Cuminum cyminum* L Essential Oil: Antioxidant, Anticancer, Antibacterial, and Antilarval Properties

**DOI:** 10.1155/2023/5075581

**Published:** 2023-02-06

**Authors:** Razie Ranjbar, Elham Zarenezhad, Abbas Abdollahi, Marjan Nasrizadeh, Samira Firooziyan, Najmeh Namdar, Mahmoud Osanloo

**Affiliations:** ^1^Department of Medical Biotechnology, School of Advanced Technologies in Medicine, Fasa University of Medical Sciences, Fasa, Iran; ^2^Noncommunicable Disease Research Center, Fasa University of Medical Sciences, Fasa, Iran; ^3^Department of Microbiology, School of Medicine, Fasa University of Medical Sciences, Fasa, Iran; ^4^Student Research Committee, Fasa University of Medical Sciences, Fasa, Iran; ^5^Urmia Health Center, Disease Control Unit, Urmia University of Medical Sciences, Urmia, Iran; ^6^Department of Tissue Engineering, School of Advanced Technologies in Medicine, Fasa University of Medical Sciences, Fasa, Iran; ^7^Department of Medical Nanotechnology, School of Advanced Technologies in Medicine, Fasa University of Medical Sciences, Fasa, Iran

## Abstract

*Cuminum cyminum* L. is a widespread medicinal plant with a broad spectrum of biological activity. In the present study, the chemical structure of its essential oil was examined utilizing GC-MS analysis (gas chromatography-mass spectrometry). Then, a nanoemulsion dosage form was prepared with a droplet size and droplet size distribution (SPAN) of 121 ± 3 nm and 0.96. After that, the dosage form of the nanogel was prepared; the nanoemulsion was gelified by the addition of 3.0% carboxymethyl cellulose. In addition, the successful loading of the essential oil into the nanoemulsion and nanogel was approved by ATR-FTIR (attenuated total reflection Fourier transform infrared) analysis. The IC50 values (half maximum inhibitory concentration) of the nanoemulsion and nanogel against A-375 human melanoma cells were 369.6 (497–335) and 127.2 (77–210) *μ*g/mL. In addition, they indicated some degrees of an antioxidant activity. Interestingly, after treatment of *Pseudomonas aeruginosa* with 5000 *µ*g/mL nanogel, bacterial growth was completely (∼100%) inhibited. In addition, the growth of *Staphylococcus aureus* after treatment with the 5000 *μ*g/ml nanoemulsion was decreased by 80%. In addition, nanoemulsion and nanogel LC50 values for *Anopheles stephensi* larvae were attained as 43.91 (31–62) and 123.9 (111–137) *µ*g/mL. Given the natural ingredients and promising efficacy, these nanodrugs can be regarded for further research against other pathogens or mosquito larvae.

## 1. Introduction

Skin cancer, with more than 1 million new cases, was the fifth most common cancer worldwide in 2020 [1]. Skin cancer is categorized into melanoma and nonmelanoma; melanoma is defined as the uncontrolled proliferation of melanocytes [2]. Melanoma, with ∼55,500 deaths annually, is the most dreadful kind of skin cancer [3]. The direct and indirect costs of treating this disease are estimated at 3.3–8.1 billion and 3.5 billion US$ in a year [4, 5].

*Staphylococcus aureus* and *Pseudomonas aeruginosa* are two important Gram-positive and Gram-negative pathogens that can bring about contagious soft tissue and skin infections with different symptoms, including color changes, pain, and swelling [6]. Besides, they could enter the body and cause severe and life-threatening infections such as septicemia [7]. Moreover, malaria, which claims about half a million lives every year, is still one of the most feared infectious diseases; it is communicated to humans via bites of infected female *Anopheles mosquitoes* [8]. Moreover, *Anopheles stephensi* Liston is among the most significant malaria vectors in South Asia and the Middle East [9].

Due to drug resistance and environmental pollution, there have been many attempts to develop plant-based medicines and insecticides [10, 11]. Essential oils (EOs) are volatile oils secreted as secondary metabolites from various parts (e.g., seeds, bark, rhizomes, and flowers) of aromatic plants [12, 13]. EOs have many biological impacts, such as antibacterial, anticancer, and insecticidal impacts [14]. For example, *Cuminum cyminum* L. (curium or jeera) is a fragrant plant that belongs to the Apiaceae family [15]. Its EO possesses antifungal effects [16], antioxidant effects [17], antibacterial effects [18, 19], and anticancer effects [20].

Despite the advantages of EOs as drugs or insecticides, such as biocompatibility and biodegradability, their effectiveness should be enhanced. The preparation of essential oil-based nanopreparations, including nanoemulsions, nanoparticles, lipid nanovesicles, and nanogels, has recently been presented as a promising method to improve the stability and efficacy of essential oils [21, 22]. In this study, nanoemulsion and nanogel containing *C. cyminum* EO were first prepared. Their biological activities were then compared, including anticancer, antioxidant, antibacterial, and larvicidal effects.

## 2. Materials and Methods

### 2.1. Materials

A-375 melanoma cells (ATCC CRL-1619), *P. aeruginosa* (ATCC 27853), and *S. aureus* (ATCC 25923) were attained from the Pasteur Institute of Iran. Urmia University of Medical Sciences (Iran) provided the required larvae (late-3rd or young-4th) of *A. stephensi*. Mosquitoes were grown and kept at a temperature of 29 ± 2°C and humidity of 70 ± 5%. The researchers in this study did not have a farm, so they bought the EO from Tabib Daru Pharmaceutical Company (Iran) with standard farms. *C. cyminum* EO was extracted from seeds. Carboxymethylcellulose (CMC), DPPH (2,2-diphenyl-1-(2,4,6-trinitrophenyl) hydrazyl), and MTT (3-(4,5-dimethyl-2-thiazolyl)-2,5-diphenyl-2H-tetrazolium) powders were purchased from Sigma-Aldrich (USA). Mueller–Hinton agar, Mueller–Hinton broth, and Tween 20 and 80 were obtained from Merck Chemicals (Germany).

### 2.2. GC-MS Analysis

EO was analyzed using a gas chromatography unit (Agilent 6890, HP-5MS column, USA) linked to a mass spectrometer (Agilent 5973, USA), as explained in our previous study [23]. In brief, the column temperature was set to 40°C (fixed for 1 minute), then ramped up to 250°C at 3°C/minute and kept at this temperature for 60 minutes. The injection port and detector temperatures were fixed at 250 and 230°C, respectively. Helium (99.999%) was utilized as a carrier gas; other operating conditions included split 25 mL/min, septum purge 6 mL/min, and column flow rate 1 mL/min. Mass spectra were obtained in the full scan mode in the 50–550 m/z range with 70 eV ionization energy. Retention indices were measured using a mixture of n-alkanes (C6–C27) employing the van den Dool and Kratz formula. EO components were determined by the combination of temperature-programmed retention indices and mass spectrometry with ADAMS and NIST 17.

### 2.3. Preparation of Nanoemulsions Containing *C. cyminum* EO

Spontaneous emulsification was utilized to prepare nanoemulsions containing *C. cyminum* EO [24]. In brief, a defined amount of *C. cyminum* EO (100 *µ*L) and various levels of Tween 20 and Tween 80 (100–300 *µ*L) were first mixed for 5 min to form a homogenous solution (2000 rpm, at room temperature). Then, distilled water was added dropwise to the desired volume (5000 *µ*L) and stirred for 40 min to stabilize (2000 rpm, room temperature).

DLS (dynamic light scattering) type apparatus was used to analyze the prepared nanoemulsions' size. Moreover, the droplet size distribution (SPAN) was measured with the equation *D*90-*D*10*/D*50, where *D* is the diameter and 90, 10, and 50 are the cumulative percentages of droplets with lower diameters than the specified values. A nanoemulsion with optimal size characteristics, including a droplet size of <200 nm and SPAN <1 [25], was selected for further investigation. Note, the optimal nanoemulsion contained 2% v/v *C. cyminum* and 5% v/v Tween 80. A similar method was applied to prepare nanoemulsion (-oil), only *C. cyminum* EO was not used.

For short-term stability analysis of the nanoemulsion, 5 mL samples were prepared and divided into five 1 mL microtubes. The three microtubes were centrifuged at −4, +4, and +25°C (14,000*g*, 30 min). Two other microtubes were used for freeze-thaw and heat-cool stability assays. In a freeze-thaw cycle, the microtube was placed at −20°C (freezer) and at room temperature for six consecutive 48-hour intervals. For the heating-cooling analysis, the microtube was stored at +45°C (steam bath) and at room temperature for six consecutive intervals of 48 hours. After each test, the samples were examined visually for sedimentation, creaming, or biphasic.

For the long-term analysis, samples were kept at 4 and 26°C for six months and then visually inspected for sedimentation, creaming, or biphasic.

### 2.4. Preparation of Nanogel Containing *C. cyminum* EO

The selected nanoemulsion was gelified by adding 3% w/v CMC; it was stirred for 4 h in a minor condition (180 rpm). The viscosity of the nanogels at shear rates of 0.1–100 1/s was examined using a rheometer machine (MCR-302, Anton Paar, Austria). The prepared nanogels were stored for six months at two temperatures of 4 and 26°C for any sedimentation or biphasic. A similar method was used to prepare nanogel (-oil), only *C. cyminum* EO was not used.

### 2.5. Investigation of Morphology of Nanoemulsion and Nanogel

TEM analysis (transmission electron microscopy) was conducted to inspect the morphology of the nanoemulsion and nanogel. One drop of each was poured on a 200-meshcarbon-coated copper grid and utilized in a TEM device (Philips EM208S 100 KV, Max Res 0.2 nm, Netherlands).

### 2.6. Investigation of Loading of the EO in the Nanoemulsion and Nanogel

ATR-FTIR analysis was conducted to examine the successful loading of the EO in the nanoemulsion and nanogel. The spectra of the EO, nanoemulsion (-oil), nanoemulsion, nanogel (-oil), and nanogels were recorded at 400–3900 cm^−1^ by means of an ATR-FTIR spectrometer (Tensor II model, Bruker Co, Germany).

### 2.7. Evaluation of Antioxidant Effects

A 0.3 mM DPPH solution (394.32 g/mol) was first prepared using absolute ethanol; 150 *µ*L/well was added to 96-well plates [26]. Then, serial dilutions of nanoemulsion and nanogel were prepared by means of absolute ethanol. By adding 50 *µ*L/well of each dilution, the antioxidant impacts of nanoemulsion and nanogel at 62.5, 125, 250, 500, and 1000 *µ*g/mL were evaluated. Then, the treated plate was incubated for 30 min in a dark connection at room temperature to complete the reaction. Finally, the absorbance of wells was read at 517 nm with the use of the plate reader (Synergy HTX multimode reader, BioTek, USA). The antioxidant effect at each concentration was measured by means of the equation A control−A sample/A control × 100. In the control group, 50 *µ*L/well of absolute ethanol was used. In the negative control groups, 50 *µ*L/well of nanoemulsion (-oil) and nanogel (-oil) was added instead of 50 *µ*L/well of nanoemulsion and nanogel (1000 *µ*g/mL); they contained equal amounts of ingredients but without *C. cyminum* EO.

### 2.8. Evaluation of Anticancer Effects

MTT assay was made to examine the cytotoxic impact of the nanoemulsion and nanogel on A-375 cells, as explained in our previous report [27]. The cells were cultured in RPMI, which contained 10% fetal bovine serum and 1% antibiotics, and seeded (1 × 10^4^ cells/well) in a 96-well plate. After the cells reached 80% confluence, the liquid content was substituted with 100 *µ*L/well of fresh media culture. Serial dilutions of nanoemulsion and nanogel were then prepared using RPMI. By adding 100 *µ*L/well of each dilution, the cytotoxic effects of nanoemulsion and nanogel at 62.5, 125, 250, 500, and 1000 *µ*g/mL were assessed. Treated plates were then incubated for 24 h at 37°C with CO_2_. After that, the liquid content was replaced with 100 *µ*L/well MTT solution (0.5 mg/mL; dissolved in RPMI), and plates were incubated in the mentioned condition for four h. Then, 100 *µ*L/well of dimethyl sulfoxide was added to dissolve the created formazan crystal. Finally, the absorbance of wells was read at 570 nm using the plate reader, and cell viability at each concentration was measured by means of the equation A sample/A control × 100. In the control group, 100 *µ*L/well RPMI was used. In the negative control groups, 100 *µ*L/well of nanoemulsion (-oil) and nanogel (-oil) was added instead of 100 *µ*L/well of nanoemulsion and nanogel (1000 *µ*g/mL); they contained equal amounts of ingredients but without *C. cyminum* EO.

### 2.9. Evaluation of the Antibacterial Activity of the Nanoemulsion and Nanogel

The ATCC100 method with a minor modification was utilized to evaluate the antibacterial activity [28]. First, *P. aeruginosa* and *S. aureus* were cultured in the Mueller–Hinton broth. Then, 2 mL of fresh bacterial suspensions (2 × 10^5^ CFU/mL) were separately poured into 5 cm plates. Then, serial dilutions of nanoemulsion and nanogel were prepared using the Mueller–Hinton broth. By adding 2 mL/plate of each dilution, the antibacterial impacts of nanoemulsion and nanogel at 1250, 2500, and 5000 *µ*g/mL were investigated. After that, treated plates were incubated at 37°C for 24 h and 10 *µ*L of each plate's supernatants were cultured on plates containing Mueller–Hinton agar (37°C, 24 h). The colonies were counted, and bacterial growth (%) was measured using the CFU sample/CFU control × 100. In the control group, 2 mL/plate Mueller–Hinton broth was utilized. In negative control groups, 2 mL/plate of nanoemulsion (-oil) and nanogel (-oil) was added instead of 2 mL/plate nanoemulsion and nanogel (1000 *µ*g/mL); they contained equal amounts of ingredients but without *C. cyminum* EO.

### 2.10. Evaluation of the Larvicidal Activity of the Nanoemulsion and Nanogel

Larvicidal bioassays were performed according to the WHO guidelines with a slight modification [29]. Beakers that contained 198.5 mL of no chlorine water, including 25 *A. stephensi* larvae, were first prepared. Serial dilutions of nanoemulsion and nanogel were then prepared using absolute ethanol. By adding 1.5 mL/baker of each dilution, the larvicidal impacts of nanoemulsion and nanogel at 12.5, 25, 50, 100, and 150 *µ*g/mL were investigated. Larval mortality after 24 h of exposure was counted. In the control group, 1.5 mL/baker absolute ethanol was used. In the negative control groups, 1.5 mL/baker of nanoemulsion (-oil) and nanogel (-oil) was added instead of 1.5 mL/baker of nanoemulsion and nanogel (150 *µ*g/mL); they contained equal amounts of ingredients but without *C. cyminum* EO.

### 2.11. Statistical Analyses

Three replicates were performed for all tests, and the final values were presented as the mean ± standard deviation. Means and standard deviations were calculated using Excel software (Microsoft Office, version 2010). Final values for all samples were compared by SPSS software using one-way ANOVA with a 95% confidence interval. The CalcuSyn software (free version, BIOSOFT, Cambridge, UK) was used to calculate the IC50 and LC50 values of the nanoemulsion and nanogels.

## 3. Results

### 3.1. Ingredients of *C*. *cyminum* EO

Eight compounds, an amount of each in EO was more than 1%, were identified using GC-MS analysis (Table 1). *β*-Pinene (11.5%), *p*-cymene (18.11%), *ɣ*-terpinene (15.3%), cuminic aldehyde (30.7%), and 1-isopropylidene-3-n-butyl-2-cyclobutene (6.62%) were the five ingredients with higher portions.

### 3.2. Prepared Nanoemulsions

**Figure 1:**
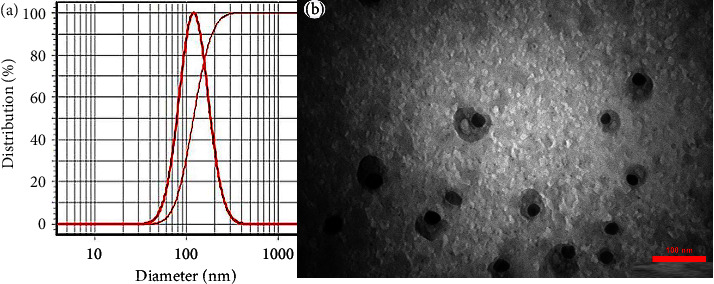
(a) Optimal nanoemulsion with a droplet size of 121 ± 3 nm and (b) TEM image (scale bar = 100 nm).

The characteristics of the size and ingredients of 10 prepared nanoemulsions are given in Table 2. Sample no. 9 with a droplet size of 121 ± 3 nm and a droplet size distribution of 0.96 showed the best size characteristics and was chosen as the optimal nanoemulsion. Its DLS profile is shown in Figure 1(a); one sharp peak also confirmed the narrow droplet size distribution. Besides, the TEM image of the optimal nanoemulsion with circular droplets is shown in Figure 1(b). In the DLS analysis, the hydrodynamic radius of the droplets is measured, so the droplet size is always larger than that of the TEM analysis. Moreover, in the DLS analysis, close droplets are identified as a droplet in concentrated systems (such as the prepared nanoemulsion).

Furthermore, no sedimentation, creaming, or phase separation was seen in the selected nanoemulsion (no. 9) after short-term (centrifugation at three temperatures, heating-cooling cycles, and free-taw cycles) and long-term (storage at two temperatures for six months) stability tests. Its stability was thus confirmed.

### 3.3. Prepared Nanogel

**Figure 2:**
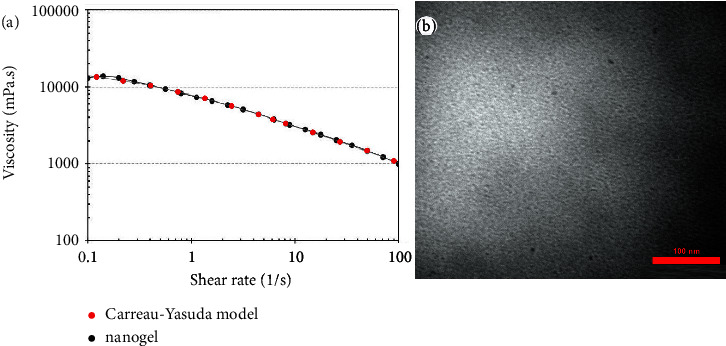
(a) Viscosity of the nanogel at different shear rates proved Carreau-Yasuda regression model and (b) TEM image of the nanogel (scale bar = 100 nm).

The optimal nanoemulsion was gelified by adding 3.0% w/v CMC. As shown in Figure 1(a), the viscosity of the nanogel almost fully fits with Carreau-Yasuda as the well-known regression of non-Newtonian fluids, i.e., the viscosity decreased with a growing shear rate [30]. Besides, the TEM image of the nanogel is shown in Figure 2(b); the nanogel droplets are not detectable due to the network structure. Furthermore, after six months of storage at 4 and 26°C, no sedimentation and biphasic were observed; nanogel's stability was thus confirmed.

### 3.4. Confirming Successful Loading of the EO

**Figure 3:**
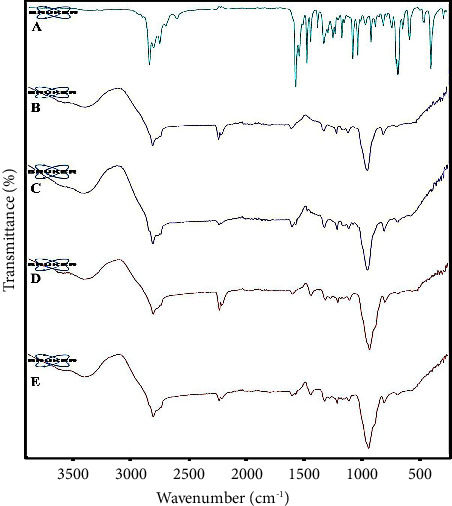
ATR-FTIR spectrums of (A) *C*. *cyminum* EO, (B) nanoemulsion (-oil), (C) nanoemulsion, (D) nanogel (-oil), and (E) nanogel.

ATR-FTIR spectra of *C. cyminum* EO, nanoemulsion (-oil), nanoemulsion, nanogel (-oil), and nanogel are shown in Figure 3. The ATR-FTIR spectrum of *C. cyminum* EO is presented in Figure 3(a); the broad peak at 3369 cm^−1^ is allocated to OH. The bands at 2960, 2925, and 2870 cm^−1^ showed the CH stretching vibration of Sp^3^ in alkanes. Besides, the bands at 2819 and 2721 cm^−1^ specify C-H aldehyde. The strong peak at 1702 and 1673 cm^−1^ corresponds to the stretching vibration of C=O in aldehyde and ketones in the EO. These strong peaks represented a high amount of aldehydes in the *C. cyminum* EO. The bands at 1575 and 1461 cm^−1^ can be related to the C=C skeleton vibration of an aromatic matter. The band at 1074 cm^−1^ was attributed to C-O stretching vibration. The band at 986 cm^−1^ corresponds to C-H bending absorption, and the strong peak at 815 cm^−1^ is allocated to the benzene ring's C-H vibration absorption. The band at 687 cm^−1^ is attributed to the vibration absorption of alkenes.

The ATR-FTIR spectrum of nanoemulsion (-oil) is displayed in Figure 3(b); the broadband between 3200–3675 cm^−1^ can be related to hydrogen bonding. The band at 2923 cm^−1^ is associated with -CH stretching vibration. Besides, the small peak at 1733 cm^−1^ can be ascribed to the carbonyl group in Tween. The band at 1251 cm^−1^ can be associated with C-OH. The main strong band at about 1085 cm^−1^ could be ascribed to C-O stretching vibration.

The ATR-FTIR spectrum of the nanoemulsion is shown in Figure 3(c); a broad and characteristic band at around 3200–3600 cm^−1^ can be ascribed to OH because of hydrogen bonding between Tween 80, water, and EO. The band at 1734 cm^−1^ is related to carbonyl stretching vibration because of EO and Tween 80. The main and strong band at 1089 cm^−1^ corresponds to C-O.

The ATR-FTIR nanogel (-oil) spectrum is displayed in Figure 3(d). The characteristic peak at 2923 cm^−1^ is ascribed to the C-H stretching vibration of alkane. The band at 1734 cm^−1^ can be related to the carbonyl group in CMC. Furthermore, the band at 1578 and 1413 cm^−1^ can be attributed to symmetric and asymmetric carboxylates in CMC. Finally, the main and strong peak at 1080 cm^−1^ can be related to C-O stretching vibration.

The ATR-FTIR spectrum of the nanogel is depicted in Figure 3(e); the broadband at about 3200–3600 cm^−1^ is attributed to the hydroxyl group owing to hydrogen bonding. This band demonstrated crosslink formation between EO, Tween 80, and CMC. The band at around 2923 cm^−1^ corresponds to C-H stretching because of EO and CMC. The peak at about 1734 cm^−1^ is similar to nanogel (-oil) and can be linked to the C=O group in CMC. The peak at 1704 cm^−1^ can be attributed to C=O stretching, representing the EO aldehyde group. Besides, the peaks at around 1575 and 1417 cm^−1^ displayed symmetric and asymmetric carboxylate groups stretching vibrations. The sharp peak at 1081 cm^−1^ confirmed the existence of C-O stretching vibration. All other peaks emerge in the spectrums of the *C. cyminum* EO and nanogel (-oil).

### 3.5. Antioxidant Effects

**Figure 4:**
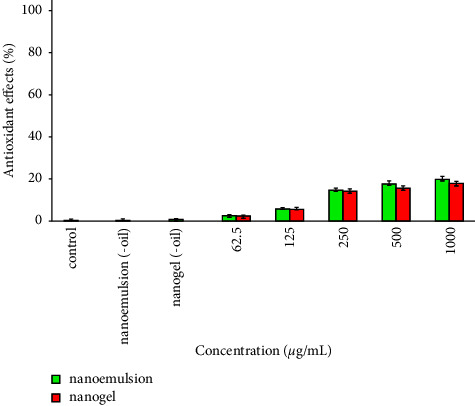
Antioxidant effects of the nanoemulsion and nanogel containing *C*. *cyminum* EO.

As shown in Figure 4, nanoemulsion and nanogel showed some degrees of antioxidant effects (3–20%). However, their efficacy was not significantly different (*P* > 0.05). Besides, nanoemulsion (-oil) and nanogel (-oil) (ingredients of nanoemulsion and nanogel 1000 *µ*g/mL) did not indicate antioxidant effects.

### 3.6. Anticancer Effects

**Figure 5:**
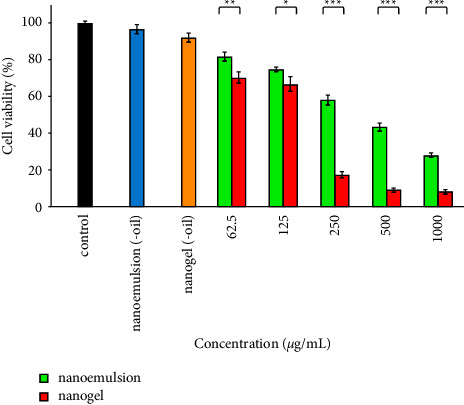
Cytotoxic impacts of the nanoemulsion and nanogel containing *C*. *cyminum* EO against A-375 cells. ^*∗*^*P* < 0.05, ^*∗∗*^*P* < 0.01, and ^*∗∗∗*^*P* < 0.001.

As presented in Figure 5, the efficacy of the nanogel was significantly more than that of nanoemulsion at all observed concentrations, including 62.5 *µ*g/mL (*P* < 0.007), 125 *µ*g/mL (*P* < 0.032), 250 *µ*g/mL (*P* < 0.0001), 500 *µ*g/mL (*P* < 0.0001), and 1000 *µ*g/mL (*P* < 0.0001). In addition, IC_50_ values of the nanogel and nanoemulsion were observed at 127.2 (77–210) *µ*g/mL and 369.6 (497–335) *µ*g/mL. Moreover, cell viability after treatment with nanoemulsion (-oil) and nanogel (-oil) (ingredients of nanoemulsion and nanogel 1000 *µ*g/mL) was decreased by 3 and 8%.

### 3.7. Antibacterial Effect

**Figure 6:**
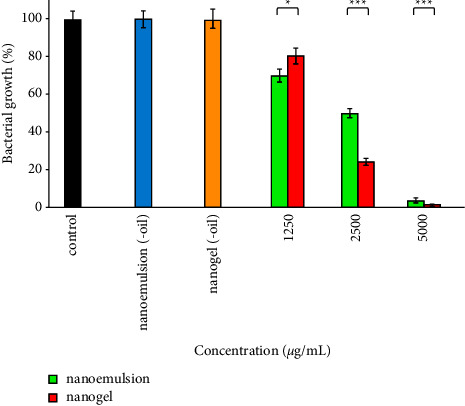
Antibacterial impacts of the nanoemulsion and nanogel containing *C*. *cyminum* EO against *P*. *aeruginosa*; ^*∗*^*P* < 0.05 and ^*∗∗∗*^*P* < 0.001.

The antibacterial activity of nanoemulsion, nanogel, nanoemulsion (-oil), and nanogel (-oil) against *P. aeruginosa* is depicted in Figure 6. The effectiveness of nanoemulsion was more than nanogel at a concentration of 1250 *µ*g/mL (*P* < 0.031). Nonetheless, the effectiveness of nanogel at two other concentrations, including 2500 and 5000 *µ*g/mL, was more effective than nanoemulsion (*P* < 0.001). Remarkably, the growth of the bacteria decreased by 100% after treatment with 5000 *µ*g/mL nanogel. Besides, bacterial growth remained unaffected after treatment with nanoemulsion (-oil) and nanogel (-oil) (ingredients of nanoemulsion and nanogel 5000 *µ*g/mL).

**Figure 7:**
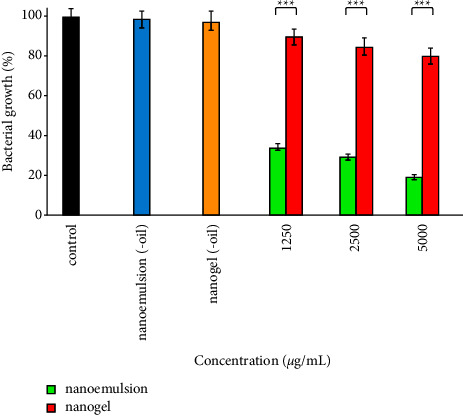
Antibacterial impacts of the nanoemulsion and nanogel containing *C*. *cyminum* EO against *S*. *aureus*; ^*∗∗∗*^*P* < 0.001.

The antibacterial activity of nanoemulsion, nanogel, nanoemulsion (-oil), and nanogel (-oil) against *S. aureus* is shown in Figure 7. The efficacy of nanoemulsion at all examined concentrations (1250, 2500, and 2500 *µ*g/mL) was remarkably more than nanogel (*P* < 0.001); bacterial growth decreased to 35, 30, and 20% after treatment with nanoemulsion. Besides, bacterial growth remained unaffected after treatment with nanoemulsion (-oil) and nanogel (-oil) (ingredients of nanoemulsion and nanogel 5000 *µ*g/mL).

### 3.8. Larvicidal Effects

**Figure 8:**
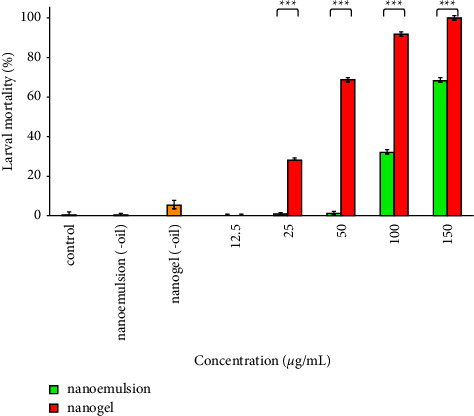
Larvicidal impacts of the nanoemulsion and nanogel containing *C. cyminum* EO against *A*. *stephensi*; ^*∗∗∗*^*P* < 0.001.

*A. stephensi* larval mortality (%) after treatment with nanoemulsion and nanogel at five concentrations (12.5, 25, 50, 100, and 150 *µ*g/mL) is illustrated in Figure 8; the effectiveness of nanogel was considerably more effective than nanoemulsion (*P* < 0.001). Moreover, LC_50_ values of nanogel and nanoemulsion were obtained as 43.91 (31–62) and 123.9 (111–137) *µ*g/mL. Interestingly, 100 and 92% larval mortality were detected after treatment with 150 and 100 *µ*g/mL nanogel. Furthermore, 0 and 5% larval mortality was seen after treatment with nanoemulsion (-oil) and nanogel (-oil) (ingredients of nanoemulsion and nanogel 150 *µ*g/mL).

## 4. Discussion

The current study used *C. cyminum* EO as a natural antioxidant, anticancer, antibacterial, and larvicidal agent. Goodarzi et al. suggested that luteolin-7-*O*-glucoside, a flavonoid in *C. cyminum*, has anticancer activities against the MCF-7 cell line (IC_50_ of 3.98 *µ*g/ml) [31]. Besides, *C. cyminum* EO showed effective antibacterial activity against *P. aeruginosa* with MIC and MBC 0.25 and 0.5 mg/mL [32]. In another study, the antibacterial activity of *C. cyminum* seeds' alcoholic extraction and EO were evaluated against *Klebsiella pneumonia*; MIC was reported at 3.41 mg/mL and 3.5 *µ*g/mL [33]. Moreover, *C. cyminum* EO showed antibacterial effects against *E. coli*, *S. aureus*, and *Listeria monocytogenes* with mean inhibition zones of 13.00, 10.00, and 17.76 mm [34].

Furthermore, some reports on nanostructures containing *C. cyminum* EO have been published. For example, the antibacterial impact of its ultrasonicated nanoemulsion was investigated against *S. aureus* in an agar diffusion well assay with an inhabitation zone of 20.3 ± 0.11 mm [35]. However, as EOs contain volatile compounds, spontaneous emulsification is preferred in such nanoformulations; droplets with the desired size and droplet size distribution are achieved by optimizing amounts of EO and surfactant (i.e., without any external energy such as ultrasonication) [36]. So, in the current study, the nanoemulsion of *C. cyminum* EO was prepared using the spontaneous method. The small droplet size permits the system to stabilize and bypass problems such as creaming or sedimentation. Besides, the low surface and interfacial tensions encourage suitable spreading and penetration of the active compounds [37]. These characteristics enhance nanoemulsions' bioavailability and efficacy [38, 39].

Despite the advantages of nanoemulsions, their topical application is challenging because of their low viscosity. In recent years, nanoemulsions-based nanogel has received more attention; they have all the benefits of nanoemulsions, and their topical application is also facilitated by enhanced viscosity [40, 41]. For instance, chitosan-caffeic acid nanogel containing *C. cyminum* EO showed more efficacy than nonformulated EO; MIC against *Aspergillus flavus* was reported at 650 and 350 ppm [42]. In another research, the enzymatic degradation in nanogel containing 5V-triphosphates was 90% less than the nonformulated state of the enzyme [43]. In the current study, the anticancer efficacy of the nanogel (IC50 127.2 µg/mL) three-folds was more potent than the nanoemulsion (369.6 *µ*g/mL).

Free radicals with unpaired electrons damage cellular membranes, lipids, proteins, and DNA directly. It also causes pivotal mechanisms causing skin aging [44, 45]. Antioxidants are substances that counteract the impacts of endogenous and exogenous oxidative stresses through scavenging free radicals. Endogenous free radicals are formed naturally through normal human metabolism; however, exogenous species result from sunlight, pollution, harsh chemicals, illness, stress, lack of sleep, and cigarette smoke [46, 47]. For topical administration of antioxidants, their stability is important. They need to be absorbed into the skin, reach their target tissue in the active form, and stay there long enough to exercise the desired impacts [48, 49]. In the current study, both nanoemulsion and nanogel showed some degrees of antioxidant effect; however, further investigation is needed.

The current study used CMC (anionic cellulose derivative) as the gelling agent, a water-soluble polymer with linear polysaccharides of anhydrous glucose [50]. Due to proper characteristics, such as mechanical resistance, viscous properties, and low cost, it has received more attention in drug delivery systems [51, 52]. This study showed that nanogel are more effective on *P. aeruginosa* than on *S. aureus*. The cell surface of *S. aureus* has a negative net charge [53]. As CMC is negatively charged, it seems proper interaction between nanogel and the bacterial membrane did not occur; however, it should be checked in future studies. On the other hand, Gram-negative bacteria such as *P. aeruginosa* have an outer membrane that envelope bacteria, improve their stability, and protect them from antibiotics [54]. However, the outer membrane is a selective permeation barrier for the hydrophilic material (i.e., nanogel) that easily enters the bacteria [55].

Excessive use of insecticides to control vector-borne diseases such as malaria has brought about environmental pollution (soil and water) and resistance against synthetic insecticides [56, 57]. Therefore, many attempts have been made to develop plant-derived insecticides, especially EO. For instance, the larvicidal effect of *C. cyminum* 0.5% crude extracts against *A. stephensi* and *Culex quinquefasciatus* was reported as 16% and 15% [58]. The present study examined the larvicidal impacts of nanoemulsion and nanogel containing *C. cyminum* EO. Interestingly, no report was found on the larvicidal impacts of a nanogel containing EO.

## 5. Conclusions

The chemical composition of *C. cyminum* EO was first identified with five compounds: *β*-pinene (11.5%), *p*-cymene (18.11%), *ɣ*-terpinene (15.3%), cuminic aldehyde (30.7%), and 1-isopropylidene-3-n-butyl-2-cyclobutene (6.62%). Nanoemulsion and nanoemulsion-based nanogel containing the EO was then prepared. Finally, a comprehensive comparison was performed on their anticancer effect (A-375), antioxidant effect, antibacterial effects (*P. aeruginosa* and *S. aureus*), and larvicidal effects (*A. stephensi*). The efficacy of nanogel against A-375 cells, *P. aeruginosa*, and *A. stephensi* was considerably more potent than the nanoemulsion. However, the nanoemulsion against *S. aureus* was more effective than nanogel. Both nanogel could be examined for more investigation against other pathogens or mosquito larvae.

## Figures and Tables

**Table 1:** Identified components in *C. cyminum* EO using GC-MS analysis.

No.	Retention time	Compound	Area	%	Retention index
1	9.427	*α*-Pinene	37379726	1.180	932
2	11.324	*β*-Pinene	367096282	11.590	979
3	13.576	*p*-Cymene	573807048	18.116	1024
4	13.668	*β*-Phellandrene	31642040	1.010	1029
5	15.18	*ɣ*-Terpinene	486212840	15.351	1059
6	23.836	Cuminic aldehyde	974724263	30.774	1239
7	25.606	1-Isopropylidene-3-n-butyl-2-cyclobutene	209723917	6.621	1271
8	33.492	*α*-Acoradiene	51304216	1.620	1464

**Table 2:** Prepared nanoemulsions: ingredients and size analyses.

No.	Ingredients (*μ*L)				Size analyses	
	*C. cyminum* EO	Tween 20	Tween 80	Water	Droplet size (nm)	SPAN^*∗*^
1	100	100	—	4800	20.3	6.25
2	100	150	—	4750	118	1.42
3	100	200	—	4700	124	1.46
4	100	250	—	4650	54.6	1.46
5	100	300	—	4600	48.8	3.31
6	100	—	100	4800	11.6	1.7
7	100	—	150	4750	40.8	1.3
8	100	—	200	4700	95.9	1.2
9	100	—	250	4650	121	0.96
10	100	—	300	4600	207	0.97

^*∗*^Droplet size distribution.

## Data Availability

The data generated or analyzed during this study are available from the corresponding author upon reasonable request.
